# Solitary myofibroma of the sigmoid colon: case report and review of the literature

**DOI:** 10.1186/1746-1596-8-90

**Published:** 2013-06-06

**Authors:** Mi-Jung Kim, Suk Hee Lee, Eui Gon Youk, Sojin Lee, Joon Hyuk Choi, Kyung-Ja Cho

**Affiliations:** 1 Departments of Pathology, Daehang Hospital, Seoul, Korea; 2 Department of Surgery, Daehang Hospital, Seoul, Korea; 3 Department of Radiology, Daehang Hospital, Seoul, Korea; 4 Department of Pathology, Yeungnam University College of Medicine, Daegu, Korea; 5 Department of Pathology, University of Ulsan College of Medicine, Asan Medical Center, 388-1 Pungnap-dong, Seoul, Songpa-gu 138-736, Korea

**Keywords:** Myofibroma, Solitary, Sigmoid colon, Adult

## Abstract

A 58-year-old woman presented with a solitary myofibroma that arose in the sigmoid colon. Computed tomography revealed a highly enhanced intramural mass (1.3-cm maximum diameter) in the proximal sigmoid colon. Histologically, the tumor exhibited a biphasic growth pattern, which comprised haphazardly arranged, interwoven fascicles of plump, myoid-appearing spindle cells with elongated nuclei and abundant eosinophilic cytoplasm, and more cellular areas of primitive-appearing polygonal cells that were arranged in a hemangiopericytomatous pattern. The tumor cells were positive for smooth muscle actin (SMA), and negative for desmin, h-caldesmon, CD34, cytokeratin, S100 protein, and CD117. The Ki-67 labeling index was not high (up to 7%). Based on these histologic and immunohistochemical features, our patient was diagnosed with a myofibroma of the sigmoid colon. The presence of solitary myofibroma in the intestine of an adult requires attention to avoid misdiagnosis as a more aggressive mesenchymal tumor.

**Virtual Slides:**

The virtual silde(s) for this article can be found here: http://www.diagnosticpathology.diagnomx.eu/vs/2096403796957687

## Background

Myofibroma, a benign neoplasm composed of myofibroblastic cells, can occur as a solitary form or as multiple or generalized form (myofibromatosis) [1]. Myofibromatosis frequently involves deeper structures and even visceral organs such as the lung, heart, gastrointestinal tract, liver, kidney, pancreas, and central nervous system [1,2]. However, solitary myofibromas usually present as a cutaneous or subcutaneous mass of the head and neck region. Solitary myofibromas involving visceral organs are extremely rare, particularly in adult patients [3-5]. We describe here a solitary myofibroma that arose in the sigmoid colon of a 58-year-old woman, with a review of the relevant literature.

## Case presentation

**Figure 1:**
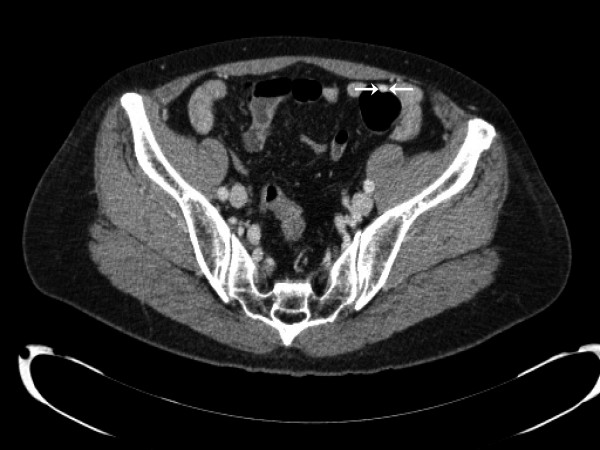
**Radiologic characterization of the lesion.** CT imaging revealed a highly enhanced intramural mass (1.3 cm in maximum diameter; white arrows) in the proximal sigmoid colon.

A 58-year-old woman, who visited our hospital for an annual check-up, presented with an incidentally found mass that had arisen in the sigmoid colon. The patient complained of intermittent abdominal discomfort, which had developed one year before admission. Five years earlier, she had experienced a stroke caused by hypertension. She had also undergone total abdominal hysterectomy due to uterine leiomyoma. Laboratory findings were unremarkable. Computed tomography revealed a highly enhanced intramural mass (1.3 cm in maximum diameter) in the proximal sigmoid colon, which was resected (Figure 1).

**Figure 2:**
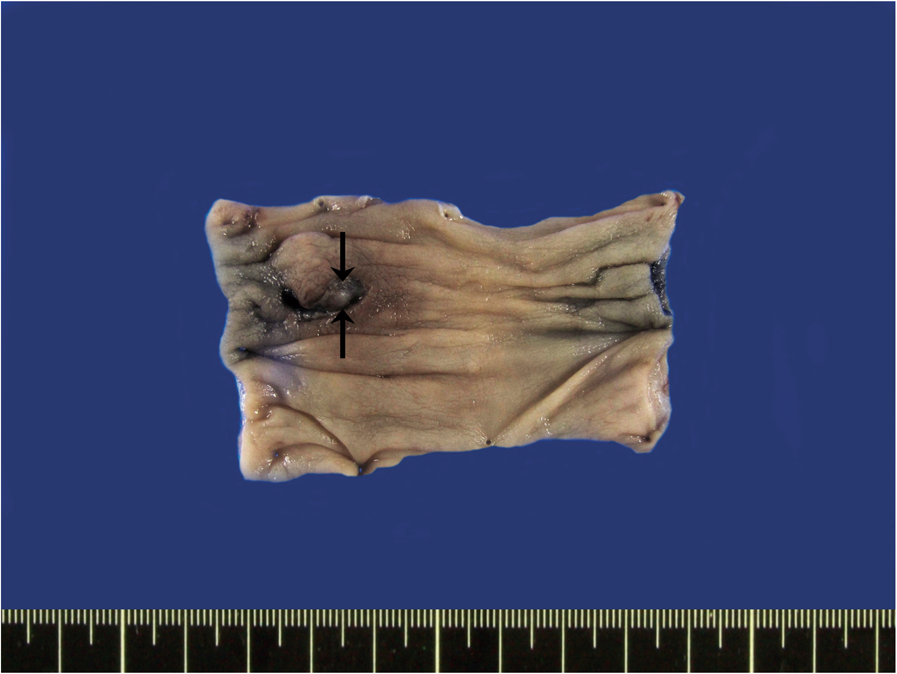
**Gross features of the lesion.** A well-demarcated, ovoid 1.3 cm × 1.0 cm × 0.7 cm intramural mass (arrows) is noted in the colon.

Gross examination showed a 1.3 cm × 1.0 cm × 0.7 cm mass with an ulcerated surface. The mass was fairly well circumscribed without encapsulation. The cut surface of the mass was homogeneously pale yellow in color, and rubbery (Figure 2).

**Figure 3:**
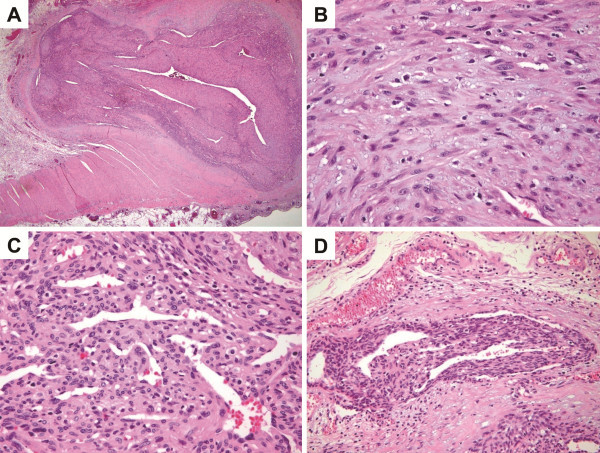
**Histologic features of the tumor. A.** The tumor is well-demarcated and situated transmurally. There are alternating light and dark areas within the tumor (H&E, ×20). **B.** The light area consists of haphazardly arranged, interweaving fascicles of plump, myoid-appearing spindle cells with elongated nuclei and abundant eosinophilic cytoplasm (H&E, ×400). **C.** The dark, more cellular area consists of primitive-appearing polygonal cells arranged in a hemangiopericytomatous pattern (H&E, ×400). **D.** Intravascular proliferation of tumor cells is noted at the periphery of the lesion (H&E, ×200).

Microscopically, the tumor was moderately cellular and consisted of both (i) haphazardly arranged, interwoven fascicles of plump, myoid-appearing spindle cells with elongated tapering nuclei and abundant eosinophilic cytoplasm, and (ii) more cellular areas of primitive-appearing polygonal cells arranged in a hemangiopericytomatous pattern (Figure 3). The myoid-appearing spindle cells blended into more cellular areas of smaller, primitive-appearing polygonal cells to impart a biphasic appearance at low magnification. The typical zonation characterized by peripheral location of the less cellular area composed of a plump, myoid-appearing, spindle cell was not evident. Intravascular proliferation of myoid-appearing spindle cells was noted at the periphery of the lesion. Mitotic figures were occasionally identified, with a frequency as high as 3 per 10 hpf. There was no evidence of necrosis, calcification or inflammatory cell components.

**Figure 4:**
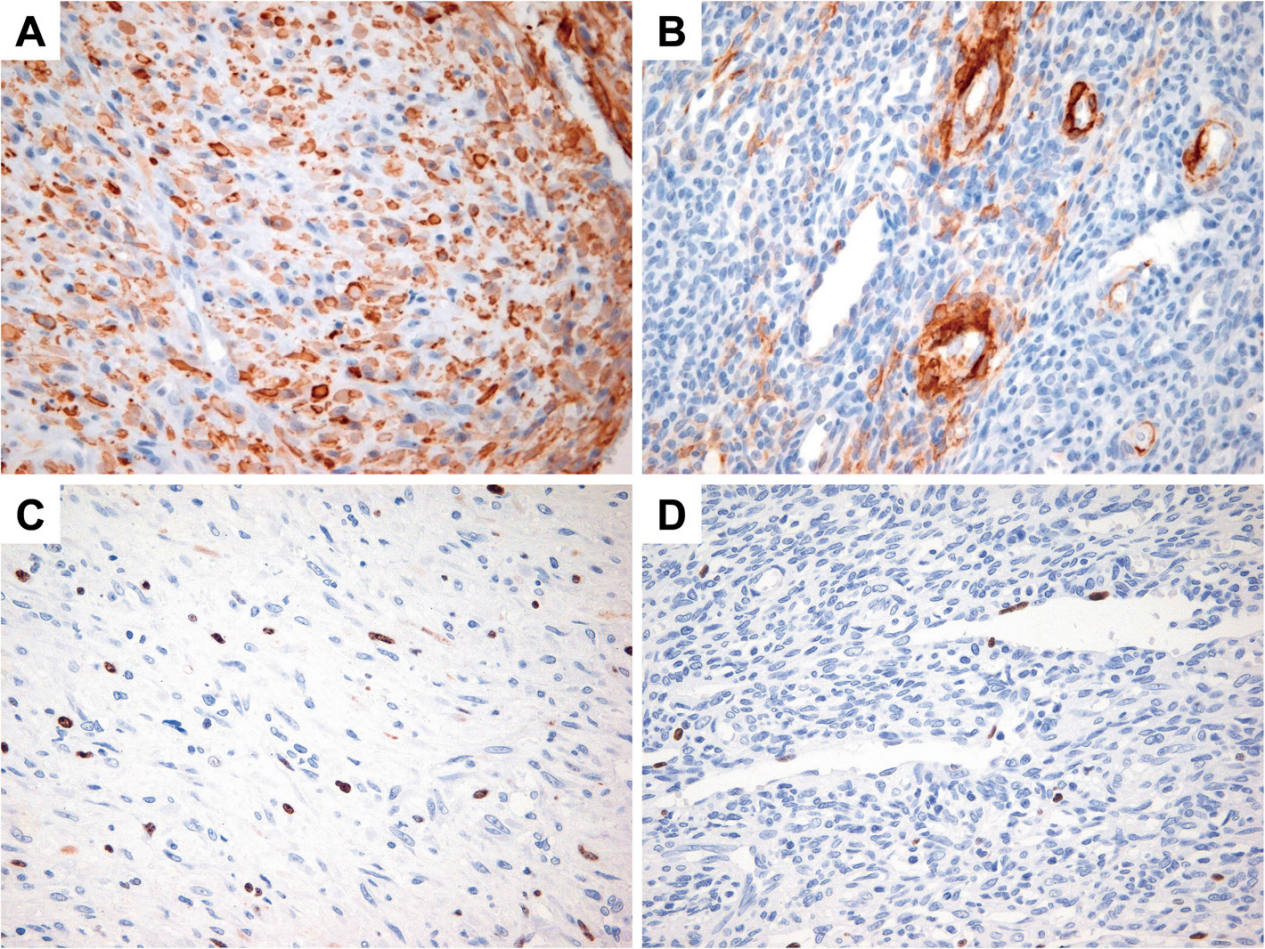
**Immunohistochemical findings. A.** The myoid-appearing spindle tumor cells show diffuse and strong positivity for SMA (immunostaining, ×400). **B.** The primitive-appearing tumor cells are focally positive for SMA (immunostaining, ×400). **C.** The myoid-appearing spindle tumor cells show an increased Ki-67 labeling index (up to 7%) (immunostaining, ×400). **D.** The primitive-appearing tumor cells display low Ki-67 labeling index (up to 1%) (immunostaining, ×400).

Immunohistochemically, the myoid-appearing spindle tumor cells were strongly positive for smooth muscle actin (SMA, 1:100; clone 1A4, Dako, Glostrup, Denmark), whereas the primitive-appearing tumor cells stained focally (Figure 4A,B). The tumor cells were negative for desmin (1:200; Cell Marque, Manchester, UK), suggesting myofibroblastic differentiation. The tumor cells were also negative for pan-cytokeratin (CK, 1:400; Novocastra, Newcastle, UK), S100 protein (1:2,000; Dako), CD117 (1:200; Dako), CD31 (1:80; Dako), CD34 (1:400; Dako) and h-caldesmon (1:100; Dako). The Ki-67 (1:100; clone 7B11, Invitrogen, UA) labeling index was not high both in the myoid-appearing spindle cells and primitive-appearing tumor cells (up to 7% in the highest area) (Figure 4C,D). Based on these histologic and immunohistochemical features, the tumor was diagnosed as a myofibroma.

The postoperative course was unremarkable, with no evidence of recurrence 17 months after surgery.

## Discussion

Myofibroma can occur as a solitary form or as multiple or generalized form (myofibromatosis) [1]. Myofibromatosis was originally described as “congenital generalized fibromatosis” in 1954 by Stout [6]. The term “infantile myofibromatosis”, which was coined by Chung and Enzinger in 1981 to distinguish it from more aggressive types of fibromatosis, reflects both the young age of onset of this disease and the myofibroblastic nature of the tumor cells [1]. Since then, the term myofibroma was introduced by Smith et al. to reflect that such lesions frequently manifest as a solitary form and affect a broad range of patients [7]. Characteristically, myofibromas are circumscribed masses with a nodular or multinodular growth pattern. At higher magnification, they show a biphasic growth pattern, consisting of fascicles or whorls of myoid-appearing spindle cells and primitive cells, arranged in a hemangiopericytoma-like pattern. The myoid-appearing spindle cells have eosinophilic cytoplasm and elongated, tapering nuclei with a vesicular chromatin, while the primitive cells have rounded nuclei and relatively scant cytoplasm with indistinct cell borders [1,2].

Despite its uniformly bland cytologic appearance, potentially worrisome histologic features can pose a great diagnostic challenge. These include fingerlike extension of tumor cells into surrounding tissue, intravascular tumor growth, hemorrhage, coagulative necrosis, and high mitotic activity (up to 10 per 10 hpf) [1,2,5]. In our case, intravascular proliferation of tumor cells was noted at the periphery of the lesion. Therefore, pathologists should be aware of the histologic spectrum of the tumors to avoid mistaken diagnosis of malignancy. For example, pleomorphic lipoma can be misdiagnosed as a nonlipomatous tumor such as myxofibrosarcoma if the tumor lacks mature fat component [8]. Similarly, solitary fibrous tumor is one of the tumors which can show a wide range of morphologic features [9]. Particularly, if solitary fibrous tumor shows epithelioid growth pattern, other tumors having epithelioid features such as epithelioid sarcoma or synovial sarcoma should be excluded.

Approximately half of solitary myofibromas occur in the cutaneous or subcutaneous tissue of the head and neck region, and the remaining half occur in deep-seated structures, such as skeletal muscle, aponeuroses, and bone. Solitary myofibromas involving the viscera are very rare, whereas up to 40% of patients with myofibromatosis have visceral lesions. Solitary myofibromas involving visceral organs have been described at various anatomic sites, including the pancreas, liver, testis, ovary, and brain parenchyma [3-5,10,11]. Most of these tumors appear within the first two years of life. The symptoms of visceral lesions are referable to the organs that are involved [2].

Intestinal involvement can be solitary or multifocal, and can involve any layer of the intestinal wall [12]. Although the gastrointestinal tract is one of the organs most frequently affected in patients with myofibromatosis, solitary gastrointestinal myofibromas are very rare, particularly after infancy [12-14]. When the tumor affects the gastrointestinal tract, the clinical manifestations are variable, with symptoms that may include diarrhea, signs of intestinal occlusion, bowel perforation, or intussusception [15]. This makes early diagnosis difficult.

Because myofibromas are extremely rare in the gastrointestinal tract, differential diagnosis to exclude other tumors is important. The differential diagnosis of myofibroma includes various types of mesenchymal tumors, including gastrointestinal stromal tumor (GIST), and tumors showing perivascular myoid differentiation, such as myopericytoma, hemangiopericytoma, glomus tumors (particularly glomangiopericytoma), and angioleiomyoma [16].

A possibility of GIST should be considered in the differential diagnosis because these are the most common mesenchymal tumors of the intestinal tract. In addition, a hemangiopericytomatous vasculature and biphasic pattern can be observed in case of GISTs [17,18]. In such cases, the use of immunohistochemistry to detect the c-kit receptor tyrosine kinase (CD117) would be helpful in diagnosis, because CD117 expression has apparently never been described for myofibromas.

Myopericytoma should also be included in the differential diagnosis. Myopericytoma forms a morphological continuum with myofibroma and can be easily confused with myofibromas [19,20]. However, in the present case, the lack of concentric perivascular proliferation of bland, round-to-ovoid cells, which is a characteristic feature of myopericytoma, favors a diagnosis of myofibroma rather than myopericytoma.

If a lesion histologically similar to myofibroma involves the gastrointestinal tract, a possibility of infantile hemangiopericytoma should be considered. Infantile hemangiopericytomas, which share histologic features found in myofibromas, are considered to arise via a morphologic continuum of the same process. Infantile hemangiopericytomas are characterized by a multilobulated growth pattern, immature-appearing neoplastic cells surrounding hemangiopericytomatous blood vessels, and plump spindle cells like myofibroma. Infantile hemangiopericytomas mostly occur in infants and frequently show increased rates of mitosis and focal necrosis [21].

Glomus tumors, particularly glomangiopericytomas, also show features of perivascular myoid differentiation. However, the tumor cells in glomangiopericytoma are uniformly rounded glomus cells with distinct cell borders, rather than plump, myoid-appearing spindle cells. Histologic features, such as the lack of myoid nodules and absence of round primitive cells in benign glomus tumors, also distinguish glomangiopericytoma from myofibroma [16]. Primary colonic glomus tumors are extremely rare, and occur almost exclusively in the stomach in the gastrointestinal tract [22,23].

Angioleiomyomas can show concentric structures of myoid cells like myopericytomas. However, the cellularity of myopericytomas is generally higher than that of angioleiomyomas, whereas the rate of desmin positivity in myopericytomas is much lower than in angioleiomyomas [24].

Whereas up to 25% of myopericytomas are desmin-positive, desmin positivity has rarely been reported in myofibromas. This suggests that myopericytomas have a myofibroblastic phenotype rather than features characteristic of smooth muscle differentiation [19,20,24]. As for h-caldesmon positivity, several studies have demonstrated that the majority of myopericytomas co-express *α*-smooth muscle actin (SMA) and h-caldesmon [20]. Expression of h-caldesmon has been reported in myofibromas, but needs to be validated in studies conducted at a larger scale [25].

Although myofibroma and myofibromatosis are defined as a benign fibroblastic/myofioblastic tumor [2], the biological behavior of the lesions is determined by the pattern of organ involvement and not by histologic features. In cases of newborns and infants with multiple visceral involvement, as many as 75% of patients die soon after birth [1,26]. However, solitary or multiple lesions confined to soft tissues and bone have an excellent prognosis. The local recurrence rates are reported to be 9% to 11% for solitary lesions. The lesions tend to undergo spontaneous regression or be cured by simple local excision. Even in cases with visceral organ involvement, the prognosis for solitary myofibromas is favorable [27]. Therefore, an excellent prognosis is anticipated in the present case, based on the solitary involvement and complete resection of the lesion.

## Conclusion

Solitary myofibromas involving the intestinal tract are extremely rare in adult patients. We here describe a solitary myofibroma arising in the sigmoid colon of a 58-year-old woman, and present a detailed review of the relevant literature. Because of its rarity and the presence of potentially worrisome histologic features, solitary adult-type myofibroma occurring in the intestine can be misdiagnosed easily, particularly when the pathologists are not familiar with this entity. Solitary adult-type myofibroma should be considered in the differential diagnosis of spindle cell tumors in the intestine and appropriate diagnosis is needed to distinguish it from malignant tumors.

## Consent

Written informed consent was obtained from the patient for publication of this Case Report and any accompanying images. A copy of the written consent is available for review by the Editor-in-Chief of this journal.

## Competing interests

The authors declare that they have no conflict of interest.

## Authors’ contributions

MJK drafted the manuscript. SHL carried out pathological examination and participated in the design of the study. EGU was responsible for the clinical data. SJL participated in the radiological analysis. JHC provided valuable insight for manuscript preparation and carried out the immunohistochemical stain evaluation. KJC revised manuscript critically for important intellectual content and had given final approval of the version to be published. All authors read and approved the final manuscript.
